# Antiviral Effect of Erdosteine in Cells Infected with Human Respiratory Viruses

**DOI:** 10.3390/pathogens14040388

**Published:** 2025-04-15

**Authors:** Pierachille Santus, Sergio Strizzi, Fiammetta Danzo, Mara Biasin, Irma Saulle, Claudia Vanetti, Marina Saad, Dejan Radovanovic, Daria Trabattoni

**Affiliations:** 1 Division of Respiratory Diseases, Ospedale L. Sacco, ASST Fatebenefratelli-Sacco, 20147 Milan, Italy; pierachille.santus@unimi.it (P.S.); fiammetta.danzo@unimi.it (F.D.); marina.saad@unimi.it (M.S.); radovanovic.dejan@asst-fbf-sacco.it (D.R.); 2 Department of Biomedical and Clinical Sciences (DIBIC), Università degli Studi di Milano, 20122 Milan, Italy; sergio.strizzi@unimi.it (S.S.); mara.biasin@unimi.it (M.B.); irma.saulle@unimi.it (I.S.); claudia.vanetti@unimi.it (C.V.); 3 Department of Pathophysiology and Transplantation, University of Milan, Via Francesco Sforza, 20122 Milan, Italy

**Keywords:** erdosteine, antiviral, oxidative stress, anti-inflammatory, SARS-CoV-2, RSV, H1N1

## Abstract

Respiratory viral infections trigger immune and inflammatory responses that can be associated with excessive oxidative stress, glutathione (GSH) depletion, and a cytokine storm that drives virus-induced cell/tissue damage and severe disease. Erdosteine is a thiol-based drug with proven mucolytic, anti-inflammatory, antioxidant, and antibacterial properties, but less is known about its antiviral effects. We performed in vitro studies to investigate the antiviral and anti-inflammatory activity of erdosteine in A549-hACE2 human lung epithelial cells infected with severe acute respiratory syndrome coronavirus 2 (SARS-CoV-2) or respiratory syncytial virus (RSV) and in Caco-2 human colon carcinoma cells infected with influenza A virus (H1N1). The cells were treated with different concentrations of erdosteine or its active metabolite 3-(4,5-dimethylthiazol-2-yl)-2,5-diphenyl-2H-tetrazolium bromide (MET-1) before and after viral infection. The viral replication/load in the cell culture supernatants was measured by real-time quantitative polymerase chain reaction (RT-qPCR) assay and digital droplet PCR. The gene expression of innate immune response signaling pathways and oxidative stress was analyzed by reverse transcription PCR custom-array. The results showed that erdosteine and its active metabolite, at concentrations consistent with an approved therapeutic human dosage, were not directly cytotoxic and had significant antiviral effects in cells pre-infected with SARS-CoV-2, RSV, and H1N1. The transcriptome analysis showed that erdosteine activated innate immune responses by stimulating overexpression of type I interferon and inflammasome pathways and modulated oxidative stress by inducing the modulation of oxidative stress and GSH pathways. These findings suggest that erdosteine may be a useful treatment for respiratory viral infections.

## 1. Introduction

Respiratory viral infections are a common cause of morbidity and mortality across all ages but especially in infants, older adults, immunocompromised individuals, or those with comorbidities [1,2,3,4,5,6,7]. Respiratory viral infections are frequently associated with acute exacerbations of chronic obstructive pulmonary disease (COPD) and asthma [8,9], with the most common respiratory viruses being rhinovirus, influenza virus, respiratory syncytial virus (RSV), parainfluenza, adenovirus, and coronaviruses [10,11]. RSV and influenza viruses are the most common causes of seasonal infections and influenza A viruses (e.g., H1N1) are responsible for pandemic influenza infections [12]. SARS-CoV-2 is the respiratory virus responsible for COrona VIrus Disease 19 (COVID-19), a highly infectious disease characterized by respiratory symptoms (e.g., fever, cough, shortness of breath, and difficulty breathing) and a diverse clinical spectrum ranging from mild to severe disease, including multiple organ failure and death [13].

During the COVID-19 pandemic, several existing and experimental drugs were tested [14]. Respiratory viruses are taken up into airway epithelial cells by receptor-mediated endocytosis and replicate in these cells, releasing viral components that are recognized by pattern recognition receptors (PRRs) [15]. Activation of the PRRs initiates a host innate immune response that induces the translocation of the nuclear factor kappa-light-chain-enhancer of activated B cells (NF-κB) and interferon regulatory factors (IRFs) to the nucleus and the transcription of proinflammatory and antiviral genes, leading to the production of various proinflammatory cytokines and chemokines, which recruit immune cells, and interferons (IFNs) that promote viral clearance and resolve the infection [15], including in the case of bacterial origin [16]. Inflammasomes are activated during viral infection and mediate the innate immune and inflammatory responses by triggering the release of proinflammatory cytokines and inflammatory programmed cell death (pyroptosis) [17]. However, in some viral infections (influenza and SARS-CoV-2), dysregulation or overactivation of the inflammasome can result in an excessive inflammatory response and a massive release of cytokines (termed “cytokine storm”), leading to persistent tissue damage and severe disease [17,18,19].

Oxidative stress is an important mechanism in the response of cells to infection with respiratory viruses [20,21,22,23]. During the excessive oxidative stress state associated with respiratory viral infections [24,25,26], an excess of reactive oxygen species (ROS) can damage cellular components, including lipids, proteins, and DNA, leading to cellular dysfunction and death [27].

Endogenous antioxidant molecules, such as glutathione (GSH), play a crucial role in mitigating oxidative stress by scavenging ROS and protecting cells from damage. Indeed, GSH is a potent non-enzymatic antioxidant in humans, supporting immune function by enhancing lymphocyte activity and cytokine production [27,28]. Moreover, GSH deficiency is common in respiratory viral infections [27,29,30], increasing the risk of oxidative damage, suggesting that maintaining or restoring its levels could be a potential therapeutic approach for these infections [26,29].

Erdosteine is a thiol agent with well-known mucolytic activity that is widely used to treat several respiratory disorders associated with thickened or increased mucus production [31]. Erdosteine has additional pharmacological properties, including anti-inflammatory, antioxidant, and antibacterial activities [32,33]. It is rapidly transformed by first-pass metabolism to the pharmacologically active metabolite N-thiodiglycolyl-homocysteine (MET-1) [32]. Given the unique pharmacological properties of erdosteine, it could be an effective treatment for patients with respiratory viral infections. Furthermore, erdosteine demonstrated to improve the quality of life and respiratory symptoms, such as dyspnea, in COVID-19 patients discharged at home [34].

In the present in vitro experiments, the potential antiviral and anti-inflammatory effects of erdosteine were examined in human cells infected with SARS-CoV-2, RSV, or influenza A virus (H1N1). We evaluated the effects of erdosteine on viral replication and the inflammatory response triggered by these viruses. We also investigated whether erdosteine could modulate innate immune responses and oxidative stress as key mechanisms known to be involved in infection with these viruses.

## 2. Materials and Methods

### 2.1. Cell Lines, Virus, and Reagents

A549 (NR-53522, human lung adenocarcinoma cells) expressing Human Angiotensin-Converting Enzyme 2 (A549-hACE2) were obtained from BEI Resources, National Institute of Allergy and Infectious Diseases (NIAID), National Institutes of Health (NIH), Bethesda, MD, USA; while Caco-2 (HTB-37™, human colon carcinoma cells, LOT #7003611) were obtained from American Type Culture Collection (ATCC®), Manassas, VA, USA. The A549-hACE2 cells were cultured in Dulbecco’s Modified Eagle Medium (DMEM) containing a high concentration of glucose (ECB20722L, Euroclone, Milan, Italy), supplemented with 10% fetal bovine serum (FBS), and 1% L-glutamine-Penicillin-Streptomycin. In the Caco-2 cells, the DMEM was supplemented with 10% FBS and 1% non-essential amino acids. The cells were maintained at 37 °C in 5% CO_2_ and at 98% humidity and trypsinized every 2–3 days. The cells were routinely checked for *mycoplasma* contamination by polymerase chain reaction (PCR) tests. The cells between passages 15 and 25 were used for the experiments. The cell lines were selected according to the viral models under investigation and because they have been well characterized for transcriptomic analysis, as previously described [35,36].

SARS-CoV-2 Virus Human 2019-nCoV (strain 2019-nCoV/Italy-INMI1, Rome, Italy) and Human Respiratory Syncytial Virus (hRSV A2001/3-12) were expanded in A549-hACE2 cells and the infectious viral particle concentration was determined using a 50% Tissue Culture Infectious Dose (TCID_50_) endpoint dilution assay as previously described [37]. Briefly, the A549-hACE2 cells were seeded onto 96-well plates (2 × 10^4^ cells/well) for 24 h and incubated with the specific virus in serial 10-fold dilutions from 10^6^ to 10^−4^ TCDI_50_/mL (50 μL) for 1 h at 37 °C in 5% CO_2_. The cells were then washed in phosphate-buffered saline (PBS) to remove unbound virus and incubated at 37 °C in 5% CO_2_ for 72 h. A viral titer from the cell supernatants was determined to assess the TCID_50_ through a single-step, real-time, quantitative Reverse Transcriptase-Polymerase Chain Reaction (RT-qPCR). The same procedure was adopted with influenza A virus subtype H1N1 (Influenza virus/A/Denver/1/1957) through expansion on Caco-2 cells.

Erdosteine and its active metabolite (MET-1) are commercial sources. They were supplied by Edmond Pharma Srl (Paderno Dugnano, Milan, Italy) as a powder and suspended in DMEM to obtain a concentration of 20 mg/mL immediately before use. An erdosteine potentiometric assay resulted with a ≥99% purity, whereas the MET-1 LC-MS assay resulted with a ≥97.5 purity. The cell lines used in this study (A549-hACE2 and Caco-2) express different levels of cytochrome P450, the enzyme fundamental for erdosteine metabolism to MET-1 [38]. MET-1 was used in the experiments with the A549-hACE2 cells as they do not express cytochrome P450, and erdosteine was used in the experiments with the Caco-2 cells as they express the optimal levels of cytochrome P450 to metabolize erdosteine to MET-1.

All the experiments involving the SARS-CoV-2, RSV, or H1N1 viruses were performed in a Biosafety Level 3 (BSL3) facility; before a sample analysis outside the BSL3 area, the virus was disabled according to institutional safety guidelines.

### 2.2. Cell Viability Assay

The cytotoxic effect of erdosteine and MET-1 was assessed using the 3-(4,5-dimethylthiazol-2-yl)-2,5-diphenyltetrazolium bromide (MTT) assay. Briefly, the A549-hACE2 or Caco-2 cells were seeded into 96-well plates at a density of 2 × 10^4^ cells/well. After 24 h, the A549-hACE2 cells were treated with different concentrations of MET-1 (100, 1000, or 2000 μg/mL), whereas the Caco-2 cells were treated with similar concentrations of erdosteine. Subsequently, the cell viability was assessed after 72 h using the MTT assay. Briefly, 30 μL of MTT solution (final concentration 0.5 mg/mL) was added to each well and incubated for 4 h at 37 °C. The supernatants were then removed and 100 μL of dimethyl sulfoxide (DMSO) was added to each well and agitated on a plate shaker for 5 min. The absorbance of each well was measured at 595 nm using a Bio-Rad automated enzyme immunoassay (EIA) analyzer (Bio-Rad Laboratories, Hercules, CA, USA). The viability of the untreated cells (control) was considered 100% and the viability of the treated cells was expressed as a percentage of the control.

### 2.3. In Vitro Viral Infection Assay and Treatment Protocols

For the viral infection assay on specific cell lines (A549-hACE2 for SARS-CoV-2 and hRSV; Caco-2 for H1N1), erdosteine was added at different concentrations either before or after infection with the selected virus at a concentration of 1.26 TCID_50_/µL. Moreover, a treatment with N-acetylcysteine (NAC) was exploited as the positive control of the antiviral effect (Supplementary Materials Figure S1).

For the pre-treatment condition, the cells were seeded at a density of 7 × 10^4^ cells/mL/well in 24-well plates and incubated at 37 °C and 5% CO_2_. After 24 h, the cells were treated in triplicate with erdosteine/MET-1 (0, 100, or 1000 µg/mL) and re-incubated for a further 24 h. The cell cultures were then incubated with the virus inoculum (1.26 TCID_50_/µL) for 1 h at 37 °C and 5% CO_2_ before being rinsed twice with warm PBS (PBS at 22 °C), replenished with DMEM, and cultured in an incubator for further processing.

For the post-treatment condition, the cells were seeded at a density of 15 × 10^4^ cells/mL/well in 24-well plates and incubated at 37 °C and 5% CO_2_. After 24 h, the cells were incubated with the virus inoculum (1.26 TCID_50_/µL) for 1 h at 37 °C and 5% CO_2_, then rinsed twice with warm PBS, replenished with DMEM, treated in triplicate with erdosteine or MET-1 (0, 100, or 1000 µg/mL), and re-incubated. For the post-treatment condition only, the cells were retreated with erdosteine or MET-1 every 24 h.

### 2.4. Antiviral Effect of Erdosteine

The SARS-CoV-2 viral load in the A549-hACE2 cell culture supernatants was quantified using an integrated real-time PCR method (C-RT-PCR) at 48 h post-infection (hpi). The total RNA was extracted from the cell culture supernatant using the Maxwell RSC Instrument with the Maxwell RSC Viral Total Nucleic Acid Purification Kit (Promega, Fitchburg, WI, USA) according to the manufacturer’s instructions. For the SARS-CoV-2 evaluation, qPCR was performed on a CFX96 (Bio-Rad, Hercules, CA, USA) using the 2019-nCoV CDC qPCR Probe Assay emergency kit (IDT, Coralville, IA, USA), which targets two regions (N1 and N2) of the nucleocapsid gene. The reactions were performed according to the following thermal profile: initial denaturation (95 °C, 10 min) followed by 45 cycles of 15 s at 95 °C (denaturation) and 1 min at 60 °C (annealing-extension). Viral copy quantification was assessed by creating a standard curve from the quantified 2019-nCoV_N positive Plasmid Control (IDT, Coralville, IA, USA).

A viral quantification by Droplet Digital PCR (ddPCR) procedure was adopted for the hRSV and H1N1 evaluations. In detail, the total RNA was extracted from 200 μL of cellular supernatant with the Promega kit following the manufacturer’s instructions. The hRSV and H1N1 genomic RNA was quantified by the One-Step RT-ddPCR Advanced Kit for Probes (Bio-Rad, Hercules, CA, USA). Briefly, 5 μL of ddPCR^TM^ Supermix for Probes (No dUTP), 900 nM primers and 250 nM probes, 15 mM dithiothreitol (DTT), 20 U/μL reverse transcriptase, 2 μL of diluted samples, and nuclease-free water were mixed in a total volume of 20 μL. Different final mixes were created by adding specific primers targeting viral sequences NS1 and HA1 for hRSV and H1N1, respectively. The RNA derived from both viruses was diluted 1:100. A total of 20 μL was mixed with Droplet Generator Oil for Probes (Bio-Rad, CA, USA) and droplets were generated with the automated QX200^TM^ droplet generator (Bio-Rad, CA, USA). The droplets were transferred to a 96-well reaction plate and heat-sealed with a pierceable sealing foil sheet (PX1, PCR plate sealer, Bio-Rad, CA, USA). PCR amplification was performed in a sealed 96-well plate using a T100 thermal cycler (Bio-Rad, CA, USA). The thermal profile used was as follows: 50 °C for 60 min for reverse transcription and 95 °C for 10 min for enzyme activation, followed by 45 cycles of 95 °C for 30 s, 55 °C for 60 s, and then 98 °C for 10 min for enzyme deactivation. The droplets were read on the QX200^TM^ droplet reader (Bio-Rad) and the reactions with less than 10,000 droplets were repeated. Concentration was expressed as copies/μL. For the ddPCR analysis, the QuantaSoft software version 1.7.4.0917 (Bio-Rad, CA, USA) was used to quantify the mRNA.

### 2.5. Transcriptome Analysis by RT-PCR Custom-Array

Cellular RNA isolation and reverse transcription (RT) into cDNA, as well as amplification and quantification through real-time qPCR were performed according to a standardized protocol [39]. At 72 h post-infection, the cells were washed in PBS and collected in 200 µL RNAzol^®^ (TEL-TEST Inc., Friendswood, TX, USA) before the RNA was extracted using the phenol-chloroform method. The RNA was dissolved in RNase-free water and quantified by the Nanodrop 2000 Instrument (1 μL, Thermo Scientific, Waltham, MA, USA). The RNA (1 μg) was purified from the genomic DNA with RNase-free DNase (RQ1 DNase; Promega) and reverse-transcribed into first-strand cDNA with Moloney murine leukemia virus reverse transcriptase along with random hexanucleotide primers, oligo dT, and dNTPs (Promega, Fitchburg, WI, USA).

The cDNA was amplified and quantified by real-time qPCR (CFX96 connect, Bio-Rad, Hercules, CA, USA) through SYBR Green PCR mix (Promega, Fitchburg, WI, USA). The innate immune response signaling pathways and oxidative stress were analyzed using an RT-PCR custom-array with the following set of 62 optimized RT-PCR primer assays (see list of abbreviations): BCL2, FADD, IL1B, NLRP3, CARD6, HLA-A, IL6, NLRP4, CASP1, ICAM1, IL8, SCAF11, CASP4, IFITM1, IRF3, TBK1, CASP8, IFITM3, IRF5, TLR4, CCL2, IFNA1, ISG20, VCAM1, CXCL2, IFNAR1, MYD88, DDX58, IL18, and NFKB1 for immune response; AHR, GPX2, MPO, PRDX5, APOE, GPX3, NOS2, PTGS2, ARTN, GPX4, NOX4, SCARA3, CAT, GSR, NUDT1, SIRT2, DUSP1, GSS, OXR1, SOD1, FOXM1, GSTP1, PRDX1, SOD2, GCLC, GUSB, PRDX2, GPX1, HMOX1, and PRDX3 for oxidative stress; and beta actin (ACTB) and glyceraldeyde-3-phosphate dehydrogenase (GAPDH) were used as the housekeeping genes.

The results for the gene expression analyses were calculated using the 2^−ΔΔCt^ method [40] and presented as the average of the relative expression units (in percentage) to an internal reference sample and normalized to the housekeeping genes (GAPDH and ACTB).

### 2.6. Cytokine Quantification by Multiplex Immunoassay

For SARS-CoV-2 infection only, the concentration of 17 inflammatory and anti-inflammatory cytokines/chemokines was assessed in cell culture supernatants collected at 72 h post-infection using magnetic bead-based immunoassays (Bio-Rad, CA, USA), according to the manufacturer’s protocol via Bio-Plex 200 technology (Bio-Rad, CA, USA). As some of the targets resulted in values above the normal range, an arbitrary value of 10,000 pg/mL was assigned, while 0 pg/mL was assigned to values below the limit of detection.

### 2.7. Statistical Analyses

The statistical analyses were performed using GraphPad Prism 9. The results are expressed as the mean ± standard error of the mean (SEM) of the indicated n values. The two-tailed Student’s *t*-test was used with a *p*-value threshold of 0.05.

## 3. Results

### 3.1. Effect of Erdosteine and MET-1 on Cell Viability

**Figure 1:**
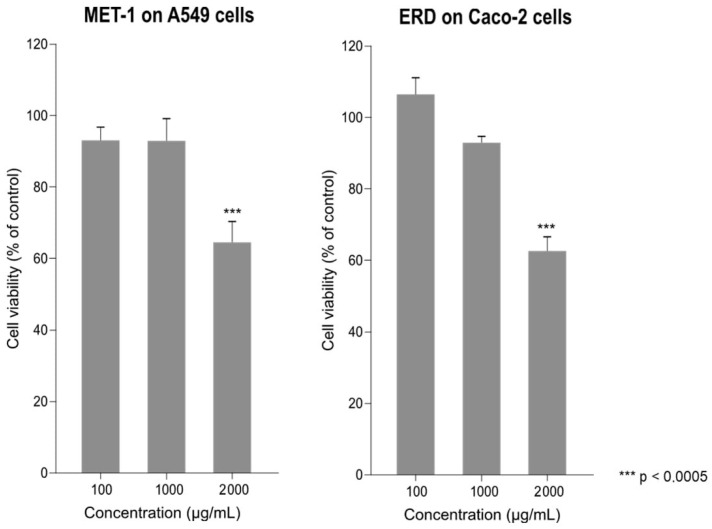
Cell viability: Effect of 100, 1000, and 2000 µg/mL of MET-1 on A549-hACE2 cell viability at 72 h post-treatment using the MTT assay (**left** panel). Effect of 100, 1000, and 2000 µg/mL of erdosteine (ERD) on Caco-2 cell viability at 72 h post-treatment using the MTT assay (**right** panel). Results are presented as mean ± SEM with each condition performed in octuplicate. Cell viability is displayed as a percentage of the viability of untreated (no drug) cells.

The effects of erdosteine and MET-1 on cell viability at 72 h post-treatment were evaluated using the MTT assay. MET-1 had no significant cytotoxic effect on the A549-hACE2 cells at concentrations of 100 and 1000 µg/mL, while there was an approximately 35% loss of cell viability at 2000 µg/mL (Figure 1). Similarly, in the Caco-2 cells, there was about a 35% loss of cell viability at the highest concentration of erdosteine (2000 µg/mL).

### 3.2. Antiviral Effects of Erdosteine and MET-1 on Virus-Infected Cells

The potential antiviral effects of erdosteine and MET-1 were evaluated by examining the effects of pre- and post-treatment with this compound in the in vitro infection assays using concentrations that did not have any direct toxicity on the cells (100 and 1000 µg/mL) when replenished every 24 h. Erdosteine and MET-1 did not demonstrate any antiviral effect when administered prior to virus infection (Supplementary Materials Figure S2).

**Figure 2:**
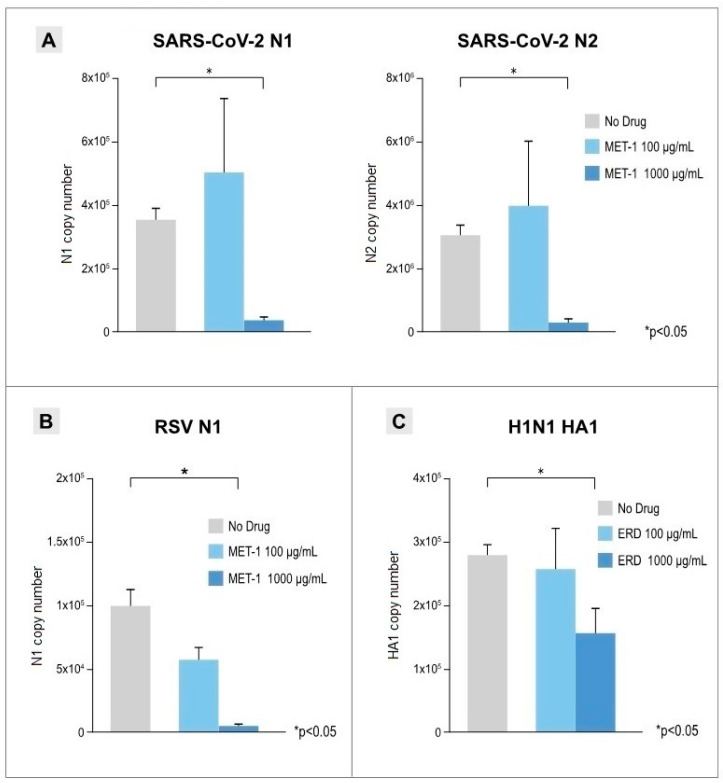
Antiviral effects of erdosteine (ERD) and its active metabolite (MET-1): (**A**) SARS-CoV-2-infected A549-hACE2 cells treated with MET-1 (100 and 1000 µg/mL). At 48 hpi, viral RNA copies in the supernatant of virus-infected cells were quantified by qPCR using two different viral targets for SARS-CoV-2: N1 (on the left) and N2 (on the right). (**B**) RSV-infected A549-hACE2 cell treated with MET-1 (100 and 1000 µg/mL). At 72 hpi, viral RNA copies in the supernatant of virus-infected cells were quantified by digital droplet PCR using a specific viral target for RSV: N1. (**C**) H1N1-infected Caco-2 cell treated with ERD (100 and 1000 µg/mL). At 48 hpi, viral RNA copies in the supernatant of virus-infected cells were quantified by digital droplet PCR using a specific viral target for H1N1: HA1. Results are presented as mean ± SEM copy number from at least 3 independent experiments, each performed in triplicate. * *p* < 0.05 vs. control (no drug).

The antiviral effects of erdosteine and MET-1 when added post-infection are shown in Figure 2. The viral load in the 48 hpi supernatant from infected cells was quantified by real-time qPCR using two different specific primers for each virus (N1 and N2 for SARS-CoV-2 sequences, RSV3 and RSV4 for RSV sequences, and HA1 and HN6 for H1N1 sequences). Erdosteine and MET-1 at the higher concentration used (1000 µg/mL) had a significant anti-viral effect compared with the control (no drug) condition in this in vitro model of SARS-CoV-2 infection of A549-hACE2 cells (91.0% inhibition; *p* < 0.05), RSV infection of A459 cells (94.5% inhibition; *p* < 0.05), and H1N1 infection of Caco-2 cells (47.5% inhibition; *p* < 0.05).

### 3.3. Immune State and Oxidative Stress Modulation Triggered by Erdosteine

**Figure 3:**
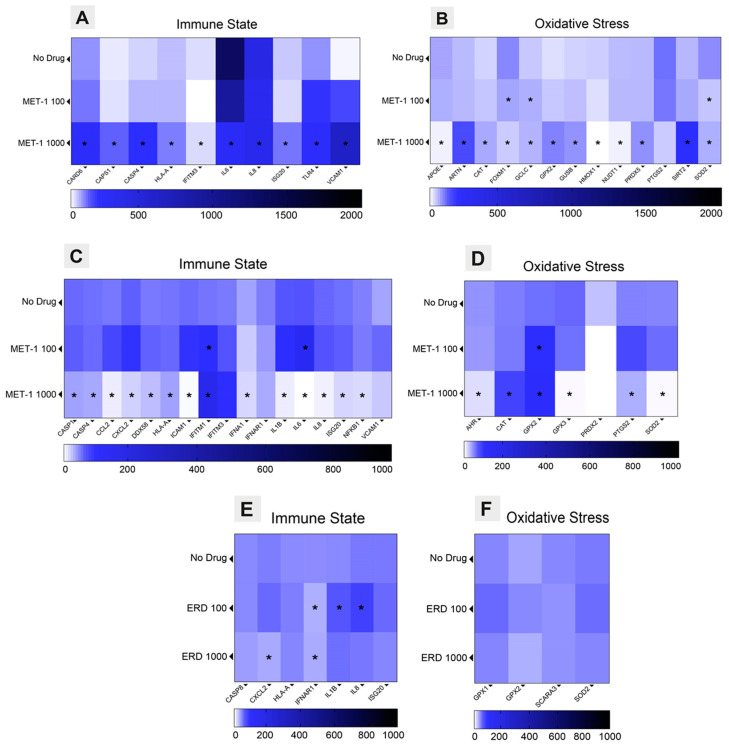
Transcriptome analysis: Heatmaps display transcriptome analysis of immune state and oxidative stress modulation by erdosteine or its active metabolite (MET-1) in SARS-CoV-2-infected A549-hACE2 cells (**A**,**B**), in RSV-infected A549-hACE2 cells (**C**,**D**), and in H1N1-infected Caco-2 cells (**E**,**F**). The units depicted on the heatmaps were obtained using the 2^−ΔΔCt^ method and presented as the average of the relative expression units to an internal reference sample and normalized to the housekeeping genes (GAPDH and ACTB). * *p* < 0.05 treated condition versus no drug.

For transcriptome characterization, cells were collected at 72 hpi, and RNA was extracted followed by reverse transcription in cDNA. Supplementary Materials Figure S4 presents a heatmap of the full dataset normalized to the GAPDH and ACTB housekeeping genes for each condition (no drug, erdosteine 100 µg/mL, and erdosteine 1000 µg/mL) in SARS-CoV-2-infected cells. Cells treated with 1000 µg/mL erdosteine showed a significant overexpression of interferon-stimulated genes (ISGs) and inflammasome components compared with the control (no drug) (Figure 3A), as well as overexpression of oxidative stress and GSH pathways (Figure 3B).

The results of the custom-array PCR for the RSV-infected cells at 72 hpi (Figure 3C,D) show that treatment with erdosteine 1000 µg/mL resulted in significantly reduced expression of numerous immune state and oxidative stress factors compared to the control (no drug). Notably, there was increased expression of the innate immune component interferon-induced transmembrane protein 1 (IFITM1) and the oxidative stress components, catalase (CAT) and glutathione peroxidase 2 (GPX2).

The custom-array PCR for the H1N1-infected cells also showed that treatment with erdosteine 1000 µg/mL significantly increased expression of immune state factors (CASP8, HLA-A, IL8, and ISG20) and decreased expression of IFNAR1 compared with no drug (Figure 3E,F). Also, there was increased expression of the oxidative stress factors GPX1, GPX2, and SOD2 with erdosteine 1000 µg/mL, while expression of SCARA3 was decreased.

### 3.4. Anti-Inflammatory Effects of Erdosteine

The concentration of 17 inflammatory and anti-inflammatory cytokines/chemokines was measured in SARS-CoV-2-infected cell culture supernatants collected at 72 hpi after treatment with erdosteine using a multiplex immunoassay. Although IL-8 and MCP-1 levels showed alterations, these changes were not statistically significant when compared to the levels in cells infected with SARS-CoV-2 alone (no drug). However, IL-5, IFN-γ, and MIP-1β demonstrated statistically significant variations in response to the treatment (Supplementary Materials Figure S3).

## 4. Discussion

This in vitro study is the first to demonstrate the antiviral activity of erdosteine against SARS-CoV-2, RSV, and influenza A virus (H1N1). Although the precise mechanisms of the antiviral effect remain to be confirmed, our results suggest that erdosteine triggers an innate immune response by stimulating type I IFN and inflammasome pathways, thereby controlling viral replication. Erdosteine also modulates oxidative stress by modulating the expression of genes involved in the oxidative stress and GSH pathways.

Erdosteine or MET-1 at concentrations that were non-toxic to A549-hACE2 or Caco-2 cells had no antiviral effects when added before virus infection but significantly decreased the viral load when added to virus-infected cells. This suggests that erdosteine does not prevent virus entry into the cells but acts intracellularly following virus entry. Consistent with this, data from the transcriptome analyses indicated that erdosteine controlled intracellular viral replication by upregulating genes involved in the innate immune response, decreasing the expression of genes for pro-inflammatory cytokines, and upregulating the expression of genes for enzymes involved in antioxidant pathways.

The innate immune system is the first line of defense against viral infection; it uses cellular and molecular strategies to recognize and remove pathogens and induces pro-inflammatory cytokines and chemokines that trigger the adaptive immune response [41]. The innate immune response to virus infection determines the clinical course and severity of disease and is initiated when PRRs detect virus-derived nucleic acids or proteins that activate antiviral and inflammatory responses, which, if excessive, can lead to deleterious systemic inflammation [42].

Respiratory viruses (SARS-CoV-2, RSV, and influenza A virus) trigger IFN responses that play a major role in early antiviral responses and limiting viral infection [43]. Type I and III IFN responses are initiated when PRRs, such as toll-like receptors (TLRs), retinoic acid-inducible gene 1 (*RIG-1*) receptors, and nucleotide-binding oligomerization domain (NOD)-like receptors, recognize pathogen-associated molecular patterns (PAMPs). This, in turn, activates IRFs in a signaling cascade and leads to expression of interferon-stimulated genes (ISGs) that encodes proteins which detect and inhibit viral replication [43].

We found that erdosteine upregulated ISGs and inflammasome components (CARD6, CASP1, CASP4, HLA-A, ISG20, and TLR4) and decreased gene expression for pro-inflammatory cytokines (IL-6 and IL-8) in SARS-CoV-2-infected cells, which would explain its antiviral effects. Erdosteine also affected the expression of genes involved in the innate immune responses in RSV- and H1N1-infected cells, although the pattern of gene expression in erdosteine-treated cells differed for each virus (RSV: CASP1. CASP4, CCL2, CXCL3, DDX58, HLA-A, ICAM1, IFITM1, IFNA1, IL1B, IL6, IL8, ISG20, and NFKB1; H1N1: CXCL2, IFNAR1, IL1B, and IL8). Previous in vitro studies demonstrated differential host transcriptional responses of human lung epithelial cells to respiratory viruses, including SARS-CoV-2, RSV, and influenza A virus, with SARS-CoV-2 eliciting a distinct pattern, especially a lower IFN signaling response but a similarly high induction of genes for pro-inflammatory cytokine/chemokine [44]. Other researchers have shown that A549-hACE2 cells respond to RSV infection with a high expression of genes for pro-inflammatory cytokines and chemokines [45]. Thus, it was not surprising that erdosteine downregulated many of these genes in RSV-infected cells. In H1N1-infected Caco-2 cells, erdosteine treatment led to the upregulation of a few ISG and inflammasome components, comparable to that seen in SARS-CoV-2-infected A549-hACE2 cells.

Inflammasomes are intracellular multiprotein complexes composed of sensors, adaptors, and effector molecules that are activated during viral infection and regulate inflammation [17]. Inflammasome activation can resolve the virus infection but uncontrolled inflammasome activity can lead to hyperinflammation and tissue damage. Viruses can also inhibit inflammasome activation to enable their replication within host cells [46]. The most comprehensively studied inflammasome activated during respiratory virus infection is NOD-like receptor family pyrin domain containing 3 (NLRP3) [47,48]. Activation and assembly of NLRP3 inflammasome releases caspase-1, which cleaves the proinflammatory cytokines IL-18 and IL-1β, which stimulate T-cells and macrophages in the innate immune response [48]. Excessive NLRP3 activation is associated with the severity of SARS-CoV-2 infection and may cause the cytokine storm [49].

GSH has recently been identified as a modulator of NLRP3 inflammasome activation; exogenous GSH and its analogues increased intracellular GSH levels and strongly inhibited assembly of the NLRP3 inflammasome complex and reduced IL-1β production [50]. GSH levels are decreased in patients with a respiratory virus infection, including SARS-CoV-2 infection, and have been associated with disease severity and death in patients with COVID-19 [27,29,51]. Thus, increasing intracellular GSH levels may provide protection against excessive inflammasome activation during respiratory virus infection. NAC is a precursor for GSH and can replenish intracellular GSH levels provided the enzymes for GSH synthesis (glutamine cysteine ligase and GSH synthase) are available [26,29,52]. A recent in vitro study showed that pre-incubation of macrophages and bronchial epithelial cells with NAC inhibited NLRP3 inflammasome activation by SARS-CoV-2 [53].

The oxidative stress associated with respiratory virus infections involves enhanced production of ROS (pro-oxidants) and reduced GSH levels (antioxidant) [54]. Respiratory viruses induce ROS-generating enzymes such as nicotinamide adenine nucleotide phosphate (NADPH) oxidase and xanthine oxidase [21]. In RSV-infected A459 cells, oxidative stress was indicated by an increase in lipid peroxidation products (8-isoprostanes, MDA, and 4-HNE) and a reduction in the GSH:GSSG ratio, together with decreased expression of the antioxidant enzymes superoxide dismutase 1 (SOD1), SOD2, and glutathione S-transferase (GST) and increased expression of SOD2 [55]. Virus-induced oxidative stress activates the transcription factor nuclear factor erythroid 2-related factor (Nrf2), which increases the expression of a range of antioxidant genes important for GSH synthesis [56].

Erdosteine is a thiol agent that has well-described anti-inflammatory and antioxidant properties [32]. We speculated that these antioxidant properties may protect virus-infected cells from oxidative damage. The antioxidant effects of erdosteine may result from its ability to scavenge intracellular ROS and prevent their damaging effects as seen in an in vitro model of oxidative stress in A549-hACE2 cells [57].

Erdosteine treatment results in increased GSH levels in the plasma [58] and in the bronchoalveolar lavage fluid of patients with chronic bronchitis [59]. Erdosteine might therefore also be able to increase GSH in respiratory virus-infected cells. In support of this, our transcriptome analysis showed that the gene encoding the antioxidant enzyme GPX2 was upregulated in erdosteine-treated cells infected by all three types of respiratory virus and that CAT was upregulated in SARS-CoV-2- and RSV-infected cells. The gene encoding PRDX5, another important antioxidant enzyme, was upregulated in SARS-CoV-2-infected A549-hACE2 cells treated with erdosteine compared to no drug treatment, as were other genes encoding the proteins/enzymes involved in modulating oxidative stress (ARTN, GCLC, GUSB, and SIRT2).

Respiratory viral and/or bacterial infections are associated with acute exacerbations of COPD or asthma and their severity [60,61]. Research has led to increased understanding of the mechanisms involved in virus-induced exacerbations of COPD/asthma and potential therapeutic approaches [62,63]. Our in vitro studies have shown that erdosteine has antiviral effects by inhibiting viral replication, modulating the immune response, and enhancing antioxidant gene expression. We can speculate that erdosteine may have a role in protecting against the effects of seasonal viral infections. Also, the antiviral effects of erdosteine, together with its antioxidant and anti-inflammatory effects, may contribute to the findings of the RESTORE study, which found that erdosteine (300 mg twice daily) as add-on therapy to usual COPD therapy for 12 months resulted in a reduced rate and duration of exacerbations in patients with moderate and severe COPD and a history of exacerbations [64,65,66].

The strengths of our study are that we determined the activity of erdosteine against several human respiratory viruses. Assessment of viral replication at 48 h for SARS-CoV-2 and H1N1 and at 72 h for RSV was based on the kinetics of infection for the different viruses. Analysis of the transcriptome and cytokines in the supernatant was standardized to 72 h for all three viruses. According to a validated model by Sainas et al. [67] that uses calculated coefficients of molarity, we determined that the concentration of erdosteine used in vitro (1000 µg/mL) is consistent with the recommended clinical dose (300 mg).

The limitations of this study are that the experiments were conducted using in vitro cell culture-based infections, which may respond differently to cells and tissues within the human body. Also, our experiments used A549-hACE2 cells and Caco-2 cells, and it is possible that other human lung cell lines (e.g., Calu-3) may have given different results. Further preclinical and clinical studies of the antiviral activity of erdosteine are needed.

## 5. Conclusions

Our studies have demonstrated that erdosteine exerts antiviral activity against SARS-CoV-2- and RSV-infected lung epithelial (A549-hACE2) cells and against influenza A (H1N1)-infected Caco-2 cells in vitro. Erdosteine inhibits viral replication by stimulating type I interferon and inflammasome pathways and by modulating expression of genes involved in the oxidative stress and GSH pathways. Further research is needed to better understand the role of erdosteine in patients with respiratory viral infections.

## Data Availability

The original contributions presented in this study are included in the article/Supplementary Materials. Further inquiries can be directed to the corresponding author(s).

## Supplementary Materials

The following supporting information can be downloaded at: https://www.mdpi.com/article/10.3390/pathogens14040388/s1, Figure S1: Antiviral effects of N-acetylcysteine (NAC); Figure S2: Antiviral effect of erdosteine active metabolite (MET-1) in pre-treatment condition; Figure S3: Multiplex Immunoassay of erdosteine active metabolite (MET-1) in SARS-CoV-2 infection Assay; Figure S4: Transcriptome analysis.

## Abbreviations

ACTB, beta actin; AHR, aryl hydrocarbon receptor; APOE, apolipoprotein E; ARTN, artemin; BCL2, B-cell CLL/lymphoma 2; CARD, caspase activation and recruitment domain; CARD6, caspase recruitment domain family/member 6; CASP1, caspase 1; CASP4, caspase 4; CASP8, caspase 8; CAT, catalase; CCL2, chemokine (C-C motif) ligand 2; CXCL2, chemokine (C-X-C motif) ligand 2; DDX58, DEAD (Asp-Glu-Ala-Asp) box polypeptide 58; DUSP1, dual-specificity phosphatase 1; FADD, Fas (TNFRSF6)-associated via death domain; FOXM1, forkhead box M1; GAPDH, glyceraldehyde-3-phosphate dehydrogenase; GCLC, glutamate-cysteine ligase/catalytic subunit; GPX, glutathione peroxidase; GPX1, glutathione peroxidase 1; GPX2, glutathione peroxidase 2; GPX3, glutathione peroxidase 3; GPX4, glutathione peroxidase 4; GSR, glutathione reductase; GSS, glutathione synthetase; GSTP1, glutathione S-transferase pi 1; GUSB, glucuronidase beta; HLA-A, major histocompatibility complex/class I/A; HMOX1, heme oxygenase 1; ICAM1, intercellular adhesion molecule 1; IFITM1, interferon-induced transmembrane protein 1; IFITM3, interferon-induced transmembrane protein 3; IFN, interferon; IFNA1, interferon alpha 1; IFNAR1, interferon (alpha, beta, and omega) receptor 1; IL, interleukin; IL1B, interleukin 1 beta; IL6, interleukin 6; IL8, interleukin 8; IL18, interleukin 18; IRF3, interferon regulatory factor 3; IRF5, interferon regulatory factor 5; ISG20, interferon-stimulated exonuclease gene 20kDa; MCP-1, monocyte chemoattractant protein-1; MET-1, active metabolite N-thiodiglycolyl-homocysteine; MIP-1β, macrophage inflammatory protein-1 beta; MPO, myeloperoxidase; MTT, 3-(4,5-dimethylthiazol-2-yl)-2,5-diphenyl-2H-tetrazolium bromide; MYD88, myeloid differentiation primary response gene (88); NAC, N-acetylcysteine; NFKB1, nuclear factor of kappa light polypeptide gene enhancer in B-cells 1; NLRP3, nucleotide-binding and oligomerization domain (NOD)-like receptor (NLR) family/pyrin domain containing 3; NLRP4, NLR family/pyrin domain containing 4; NOS2, nitric oxide synthase 2; NOX4, nicotinamide adenine dinucleotide phosphate (NADPH) oxidase 4; NUDT1, nudix-type motif 1; OXR1, oxidation resistance 1; PRDX, peroxiredoxin; PRDX1, peroxiredoxin 1; PRDX2, peroxiredoxin 2; PRDX3, peroxiredoxin 3; PRDX5, peroxiredoxin 5; PRRs, pattern recognition receptors; PTGS2, prostaglandin-endoperoxide synthase 2; RSV, respiratory syncytial virus; SARS-CoV-1, severe acute respiratory syndrome coronavirus 2; SCAF11, Serine Arginine (SR)-related C-terminal heptapeptide repeat domain (CTD)-associated factor 11; SCARA3, scavenger receptor class A/member 3; SIRT2, sirtuin 2; SOD1, superoxide dismutase 1; SOD2, superoxide dismutase 2; TBK1, TANK-binding kinase 1; TLR4, toll-like receptor 4; VCAM1, vascular cell adhesion molecule 1.
