# Supplementary material for: Antiviral Effect of Erdosteine in Cells Infected with Human Respiratory Viruses
Source: Pathogens. 2025 Apr 15;14(4):388. doi: 10.3390/pathogens14040388 (PMC12030430; doi:10.3390/pathogens14040388)
Supplement: Supplementary file 1 [file pathogens-14-00388-s001.zip › Supplementary Files.docx]

**Supplementary Figure S1**

**Sup. Fig. S1.** **Antiviral effects of N-acetylcysteine (NAC):** SARS-CoV-2-infected A549-hACE2 cells treated with NAC (100 and 1000 µg/mL). At 48 hpi, viral RNA copies in the supernatant of virus-infected cells were quantified by RT-qPCR using two different viral targets for SARS-CoV-2: N1 (on the left) and N2 (on the right).


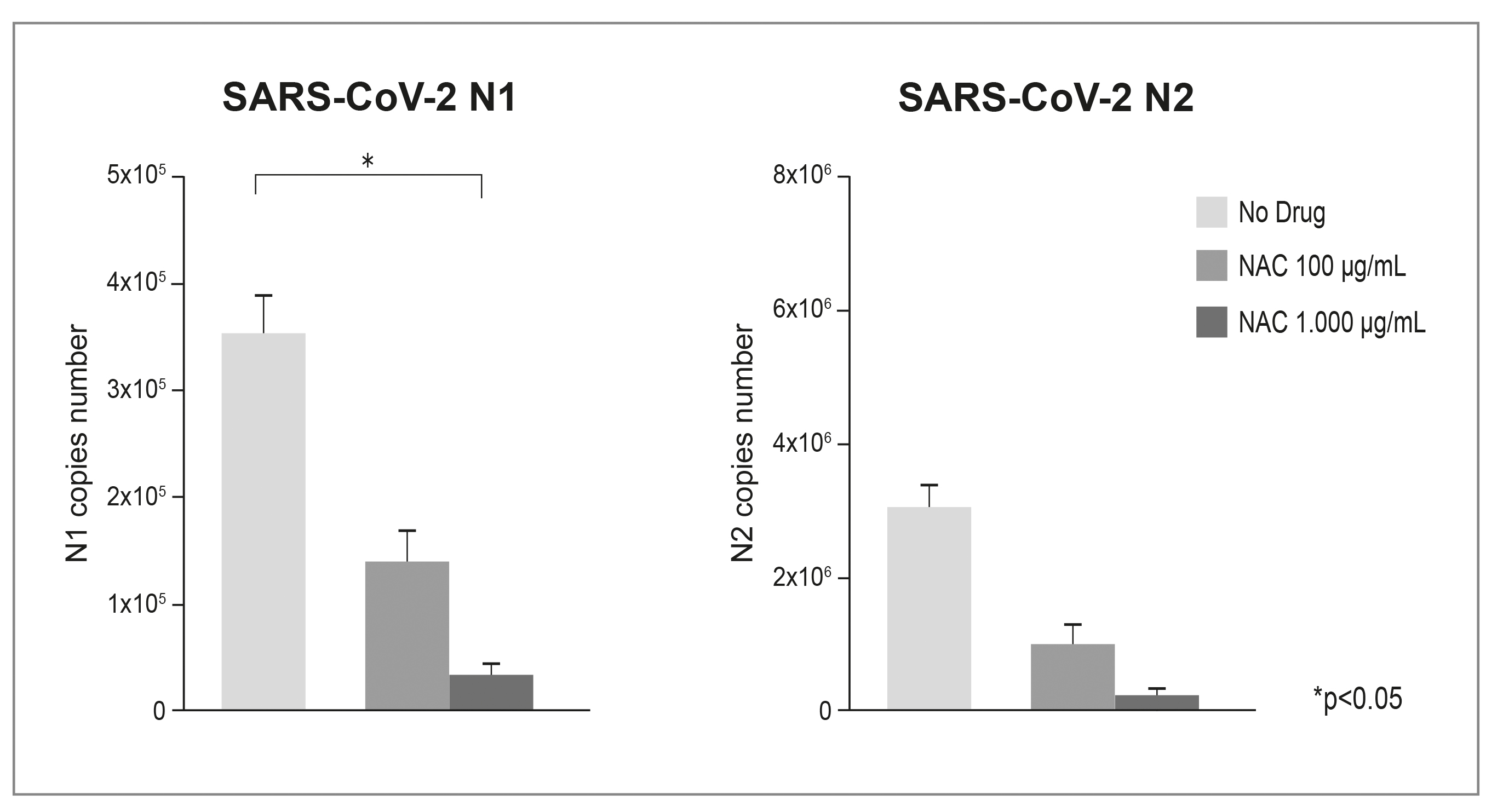

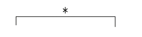


Results are presented as mean ± SEM copy number from at least 3 independent experiments, each performed in triplicate.  *p < 0.05 vs. control (No Drug).

**Supplementary Figure S2**


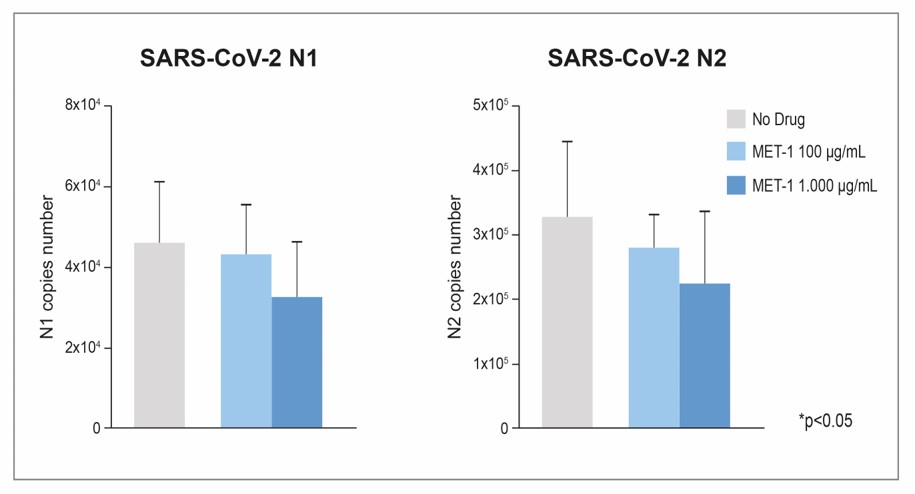


**Sup. Fig. S2.** **Antiviral effect of erdosteine active metabolite (MET-1) in pre-treatment condition: (A)** A549-hACE2 cells pre-treated with MET-1 (100 and 1000 µg/mL). After 24 h, A549-hACE2 cells were infected with SARS-CoV-2. At 48 hpi, viral RNA copies in the supernatant of virus-infected cells were quantified by RT-qPCR using two different viral targets for SARS-CoV-2: N1 (on the left) and N2 (on the right). Results are presented as mean ± SEM copy number from at least 3 independent experiments, each performed in triplicate.  *p < 0.05 vs. control (No Drug).

**Supplementary Figure S3**


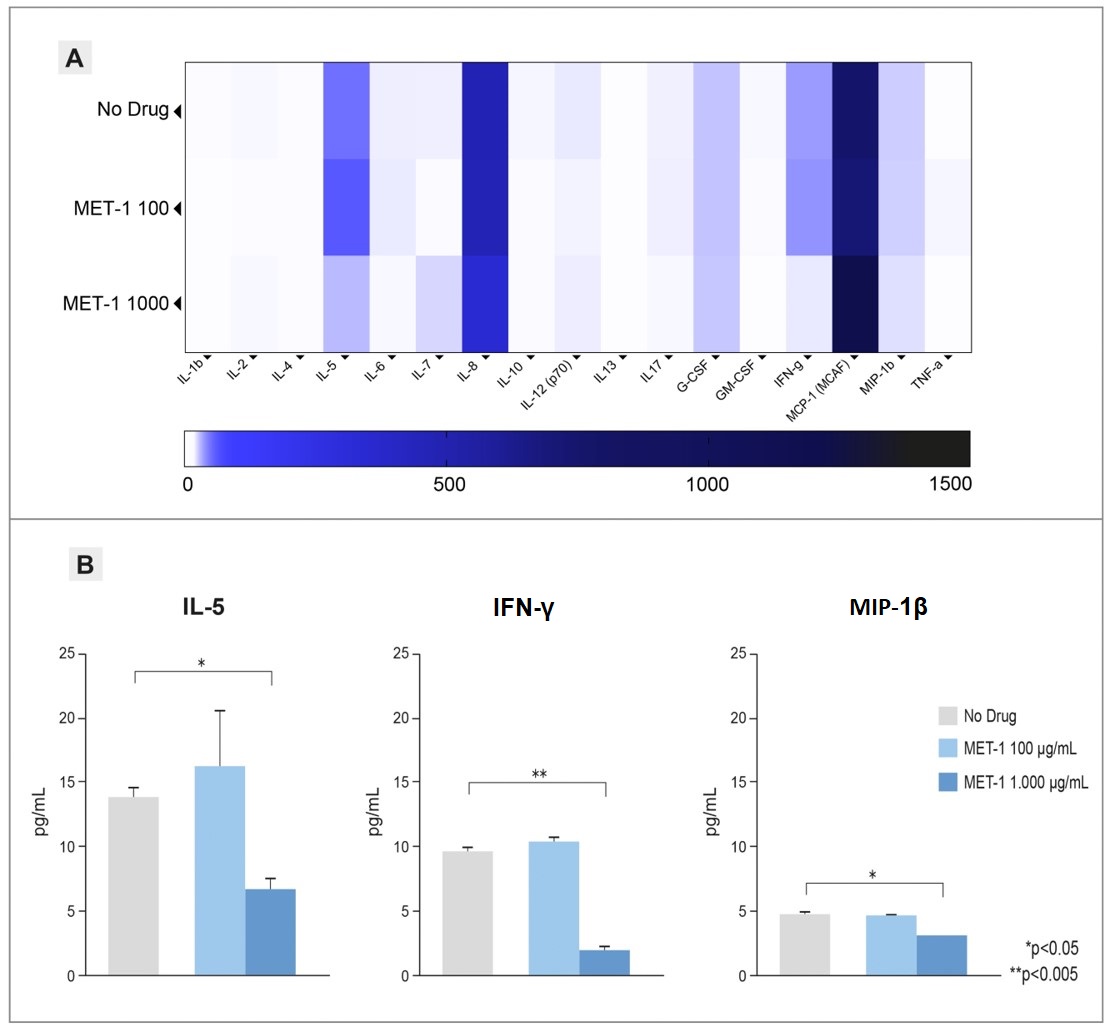


**Sup. Fig. S3. Multiplex Immunoassay of erdosteine active metabolite (MET-1) in SARS-CoV-2 infection Assay:** SARS-CoV-2 infected A549-hACE2 cells were treated with MET-1 (100 and 1000 µg/mL). At 72 hpi, 17 inflammatory and anti-inflammatory cytokines/chemokines were assessed in cell culture supernatants by Multiplex Immunoassay **(A)**. IL-5, IFN-γ, MIP-1β displayed a statistically significant variation in 1000 µg/mL MET-1-treated condition **(B)**. Results are presented as pg/mL mean ± SEM copy number from at least 3 independent experiments, each performed in triplicate.  *p < 0.05 vs. control (No Drug).

**Supplementary Figure S4**


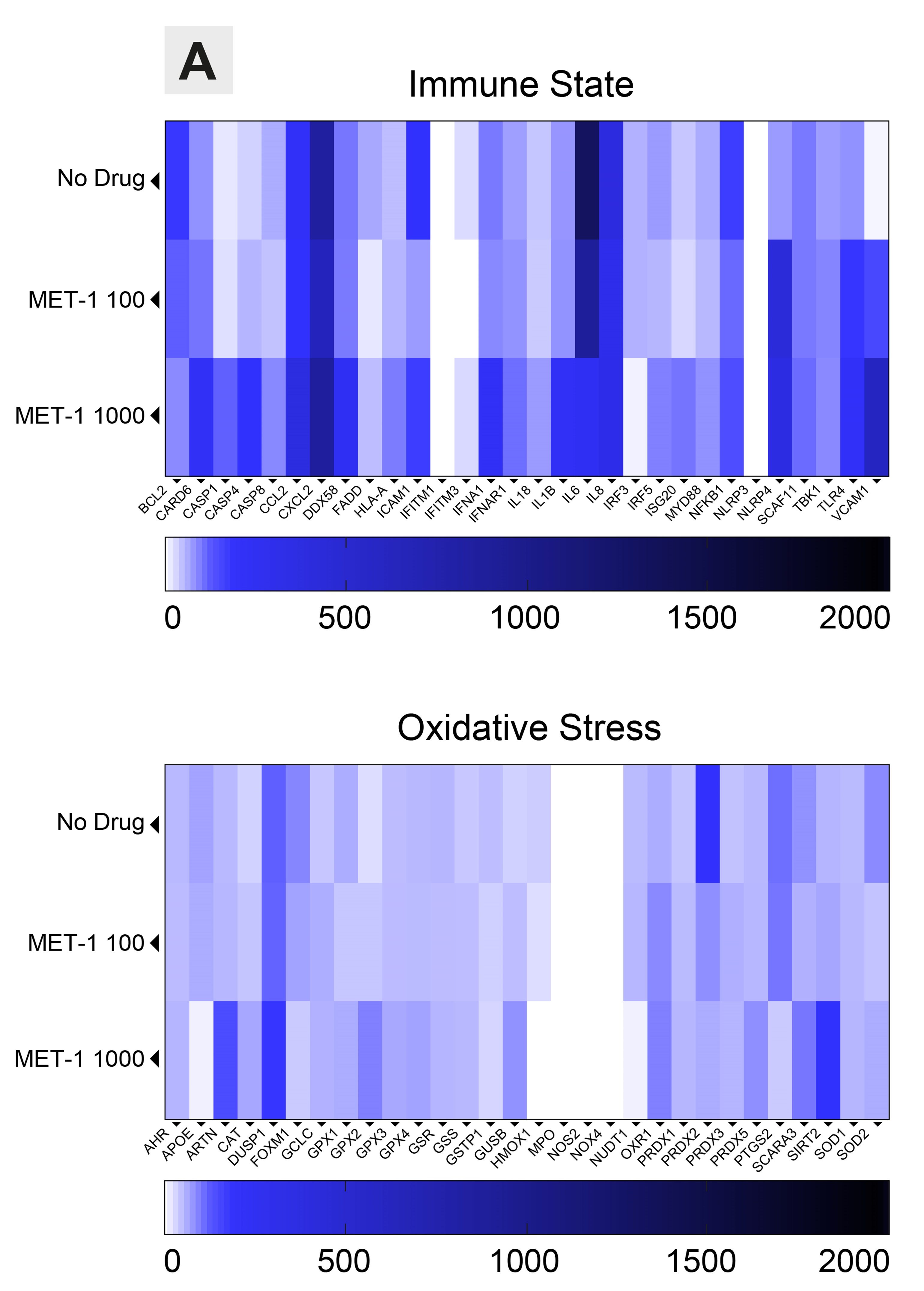


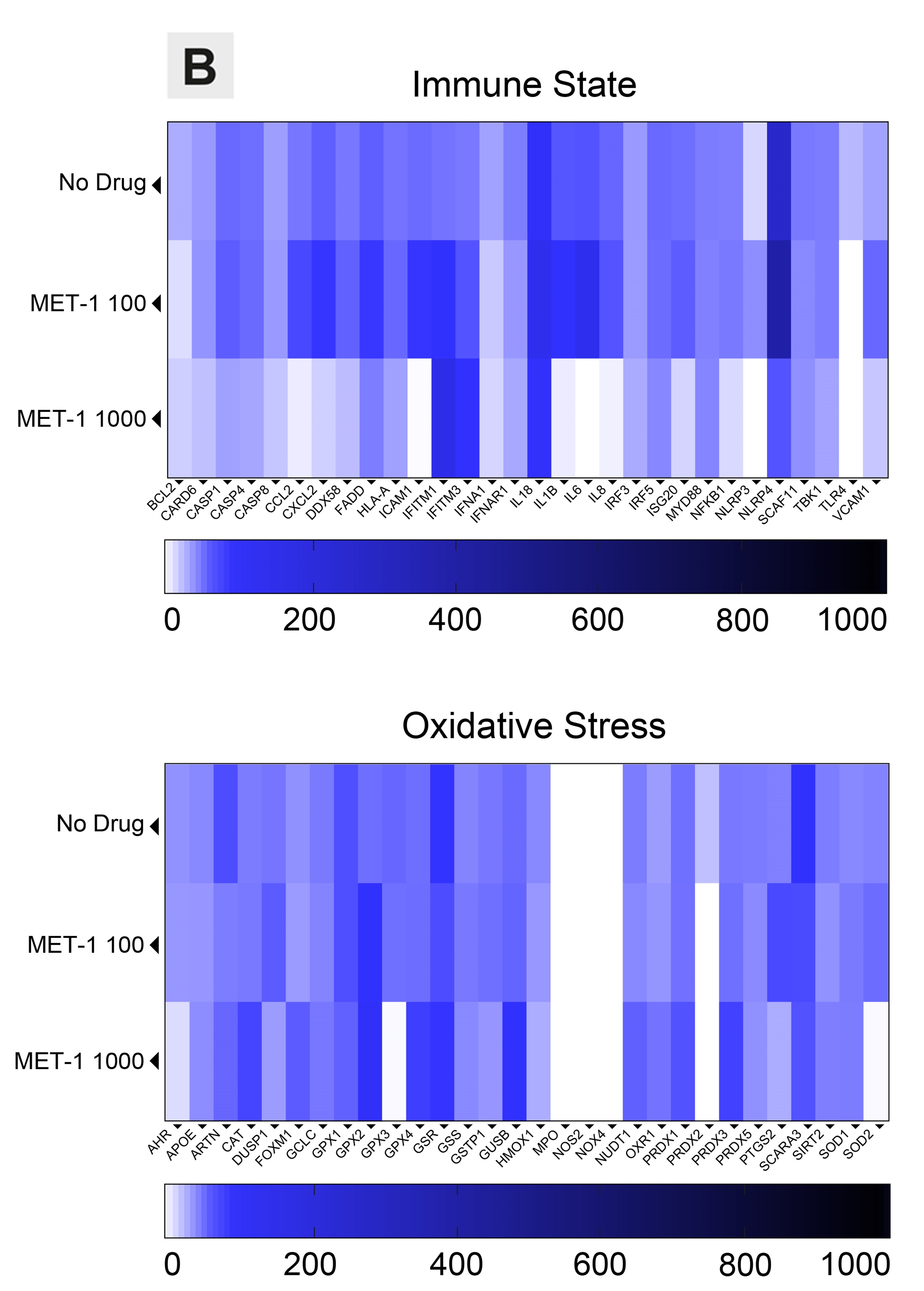


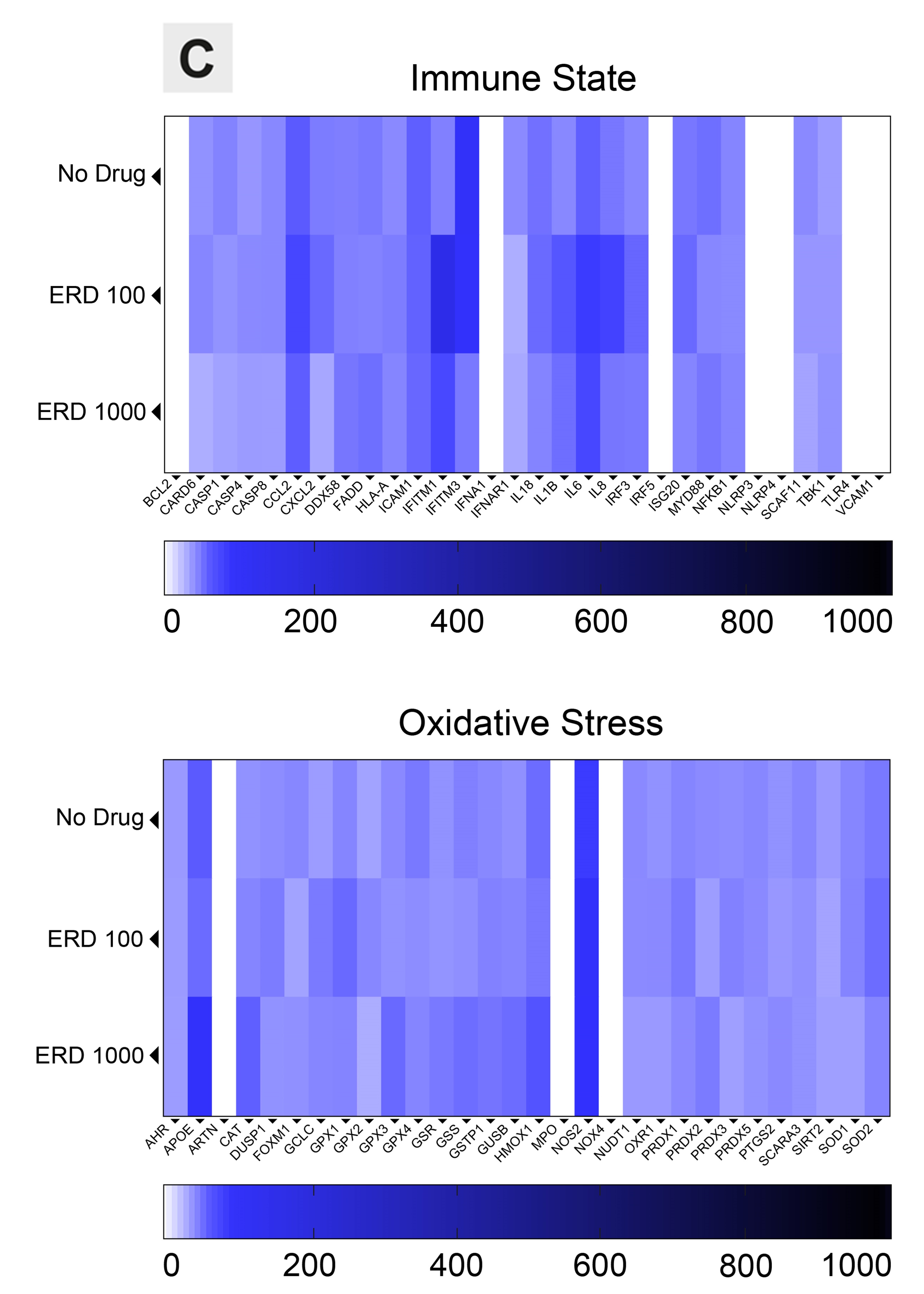


**Sup. Fig. S4. Transcriptome analysis**: Heatmaps display transcriptome analysis of immune state (above) and oxidative stress (below) modulation by erdosteine or its active metabolite (MET-1) in SARS-CoV-2 infected A549-hACE2 cells (Panel A), in RSV infected A549-hACE2 cells (Panel B); and in H1N1 infected Caco-2 cells (Panel C). The units depicted on the heatmaps were obtained using the 2−ΔΔCt method and presented as the average of the relative expression units to an internal reference sample and normalized to the housekeeping genes (GAPDH, ACTB).
