# Supplementary figures and images for: Antiviral Effect of Erdosteine in Cells Infected with Human Respiratory Viruses
Source: Pathogens. 2025 Apr 15;14(4):388. doi: 10.3390/pathogens14040388 (PMC12030430; doi:10.3390/pathogens14040388)

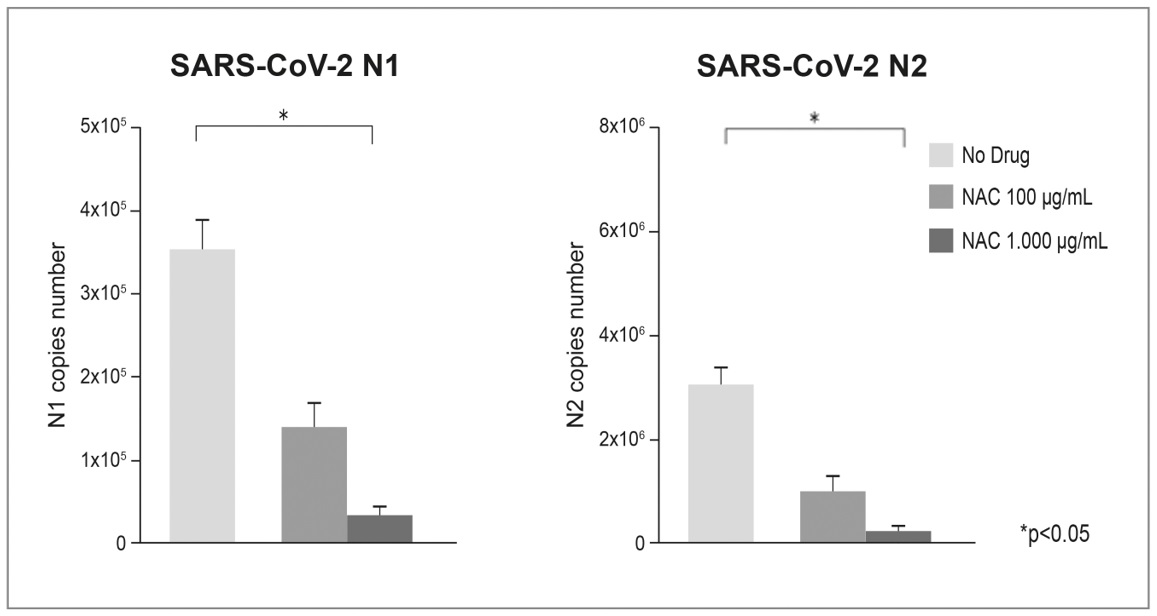

Supplement: Supplementary file 1 [file pathogens-14-00388-s001.zip › Supplementary Figure S1.jpg]

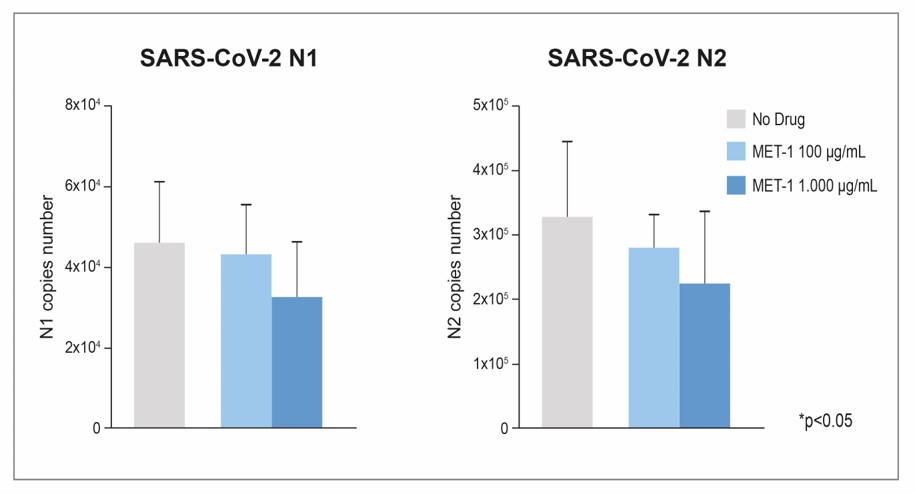

Supplement: Supplementary file 1 [file pathogens-14-00388-s001.zip › Supplementary Figure S2.jpg]

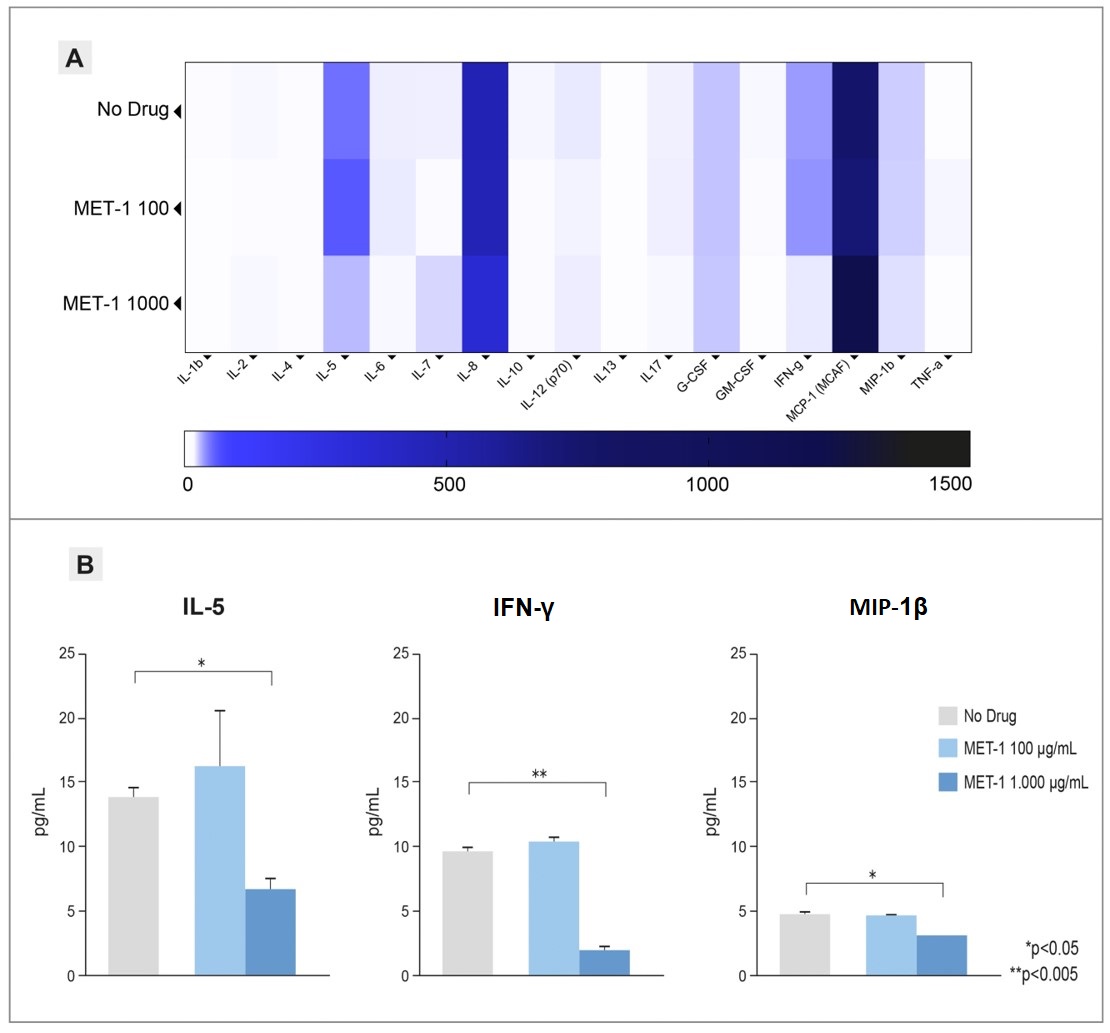

Supplement: Supplementary file 1 [file pathogens-14-00388-s001.zip › Supplementary Figure S3.jpg]

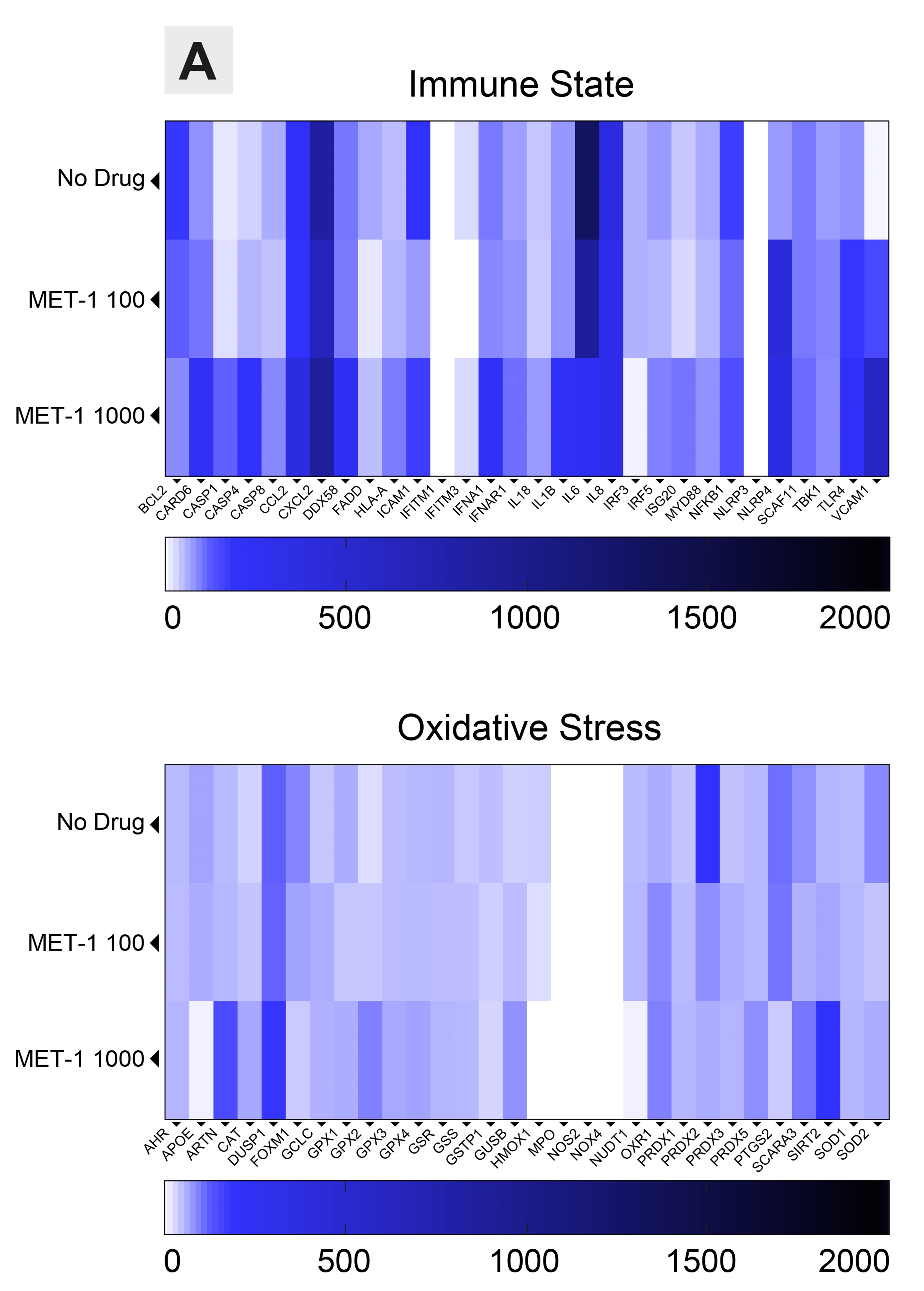

Supplement: Supplementary file 1 [file pathogens-14-00388-s001.zip › Supplementary Figure S4A.jpg]

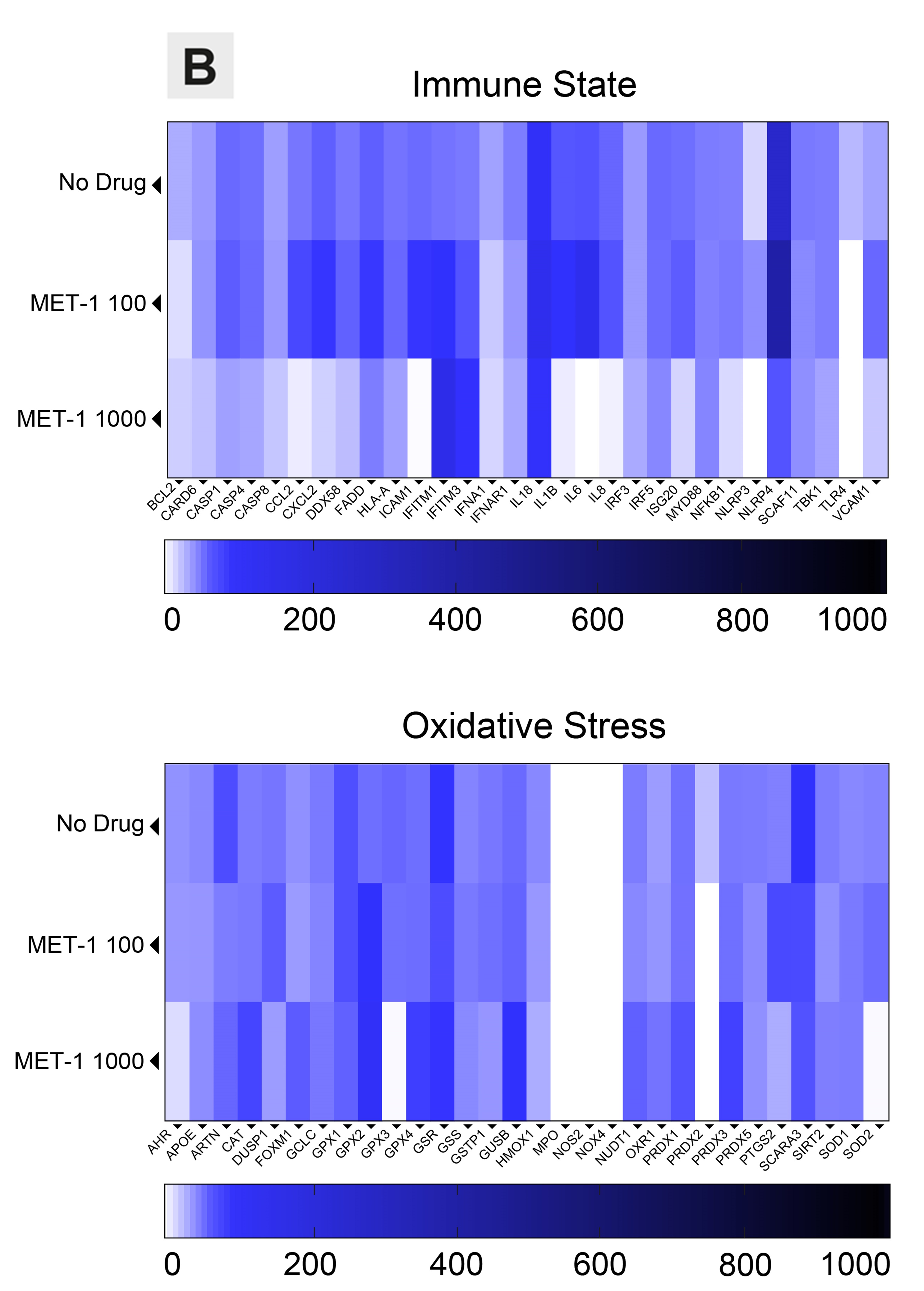

Supplement: Supplementary file 1 [file pathogens-14-00388-s001.zip › Supplementary Figure S4B.jpg]

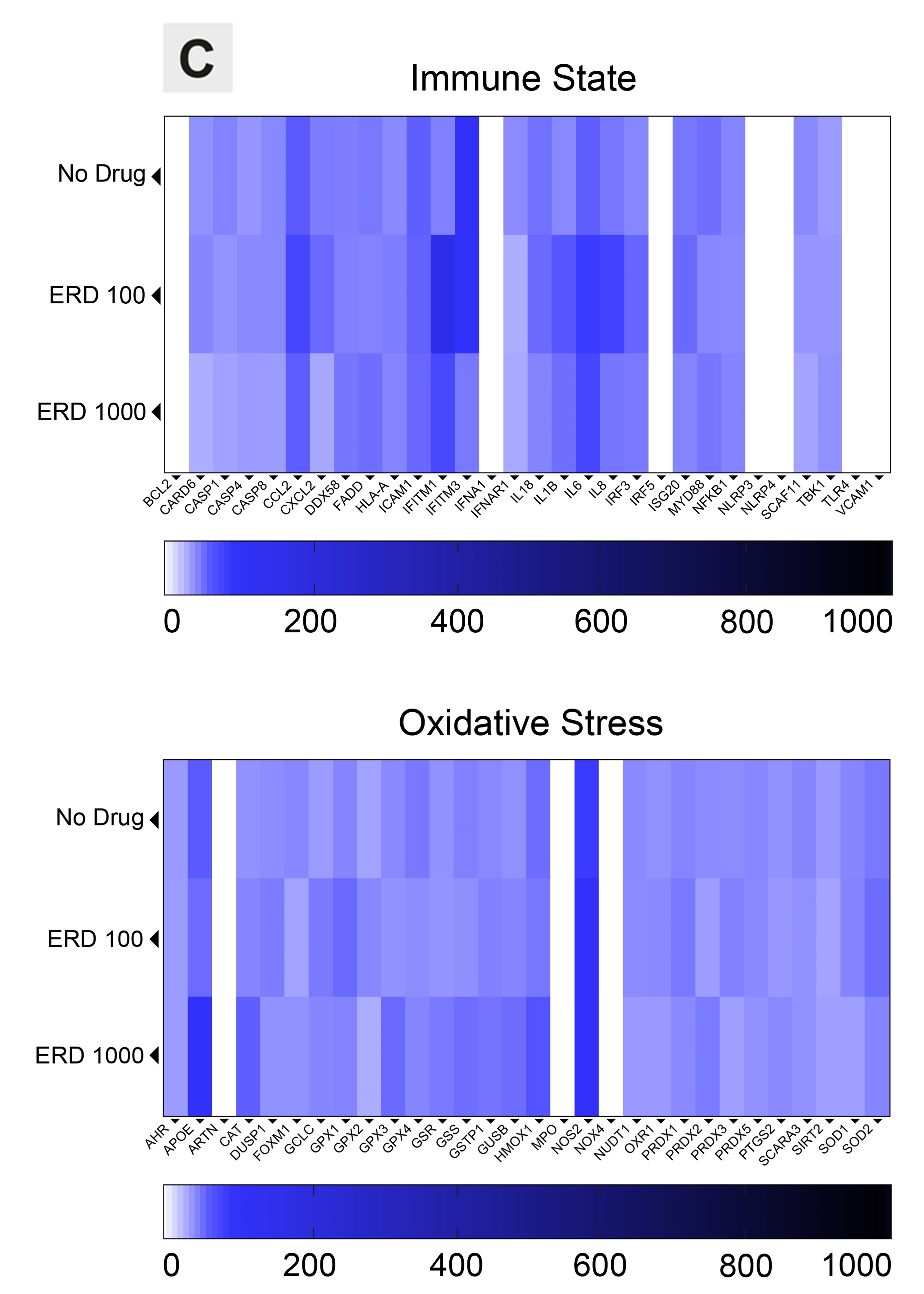

Supplement: Supplementary file 1 [file pathogens-14-00388-s001.zip › Supplementary Figure S4C.jpg]
